# Trans-Cinnamaldehyde Primes More Robust Channel Catfish Immune Responses to *Edwardsiella ictaluri* Infection

**DOI:** 10.3390/pathogens13040310

**Published:** 2024-04-11

**Authors:** Reshma Ramachandran, Emerald Ford, Basant Gomaa, Hossam Abdelhamed

**Affiliations:** Department of Comparative Biomedical Sciences, College of Veterinary Medicine, Mississippi State University, Starkville, MS 39762, USA; reshma-ramachandran@idexx.com (R.R.); emeraldford@gmail.com (E.F.); bmg381@msstate.edu (B.G.)

**Keywords:** innate immunity, adaptive immunity, pro-inflammatory genes, enteric septicemia of catfish, real-time PCR

## Abstract

Infection with *Edwardsiella ictaluri,* a causative agent of enteric septicemia of catfish, threatens profitable catfish production through inventory losses. We previously demonstrated that trans-cinnamaldehyde (TC) enhances the survival of catfish following *E. ictaluri* infection. The present study was conducted to investigate catfish immune responses to TC feeding and *E. ictaluri* infection. The expression of 13 proinflammatory, innate, and adaptive immune-related genes was evaluated over time in two sets of experiments using real-time polymerase chain reaction (PCR). In the first experiment, catfish were fed a basal diet with or without TC supplementation, while in the second they were fed a TC-supplemented or normal diet followed by infection with *E. ictaluri*. The catfish group infected with *E. ictaluri* and fed a TC-diet showed significant changes in the expression of innate and adaptive immune-related genes compared to control group. At 21 and 28 days post-infection, recovered fish showed significant increases in the expression of IgM in the anterior kidney and spleen. These results suggest that the supplemental dietary intake of TC can improve the immune status of catfish via engaging innate and adaptive immune responses and the production of memory cells in immunocompetent tissues. Together, this study provides an important foundation for the potential application of TC as an antimicrobial alternative in aquaculture.

## 1. Introduction

In the United States, the production of pond-raised channel catfish (*Ictalurus punctatus*) has the greatest economic value of any aquaculture industry at USD 437 million in revenue in 2023 alone, and the majority of this originates from southeastern states, including Mississippi [1]. While catfish farmers seek to maximize production yields, diseases caused by bacterial pathogens are responsible for high pond mortality rates and financial losses [2]. The most prominent bacterial diseases are enteric septicemia of catfish (ESC) caused by *Edwardsiella ictaluri*, columnaris disease caused by *Flavobacterium covae*, and motile *Aeromonas* septicemia (MAS) caused by virulent strains of *Aeromonas hydrophila* [3]. Each of these pathogens accounts for significant mortality and economic losses due to interruptions to production as a consequence of lost feeding time (growth) and the need for mitigation through chemical or antibiotic treatment [4,5,6].

The strategy of management for bacterial infections often rely on feed medicated with antimicrobials, including sulfadimethoxine–ormetoprim, tetracycline, and florfenicol. Other approaches include withholding feed to limit the disease transmission [7]. However, this method has some limitations as this management strategy can reduce the growth of catfish during production [8]. In addition, medicated feeds are expensive (about USD 600/ton more than traditional feeds) [9], and their utility is limited by the emergence of antimicrobial-resistant bacterial strains such that these efforts to control bacterial infections may be unsuccessful [10]. Researchers have recently been focused on developing vaccines and applying plant extracts such as aromatic plants, herbal products, and essential oils as alternative methods for controlling bacterial diseases in aquaculture settings with the goal of reducing or replacing the need for antimicrobial use [8].

Trans-cinnamaldehyde (TC) is a plant-derived phytophenolic organic compound that has recently emerged as a promising alternative to antimicrobials in animal production. TC is known to be effective against a multitude of pathogens, including bacteria [11,12], fungi [13,14], and viruses [15,16]. In general, plant-derived organic compounds can effectively kill bacteria through multiple mechanisms of action, thereby preventing bacteria from developing resistance [17]. A previous study by our group showed that the growth of *E. ictaluri* 93–146, *F. columnare* 94–081, and *A. hydrophila* ML09–119 was inhibited completely by incubation with TC at 40, 20, and 80 μg/mL, respectively. Moreover, the incorporation of TC into catfish feed significantly reduced the mortality caused by *E. ictaluri* infection and effectively minimized the bacterial colonization of catfish internal organs [12]. Functional feed additives support aquatic animals’ natural defensive mechanisms through two mechanisms: (i) direct activation of the innate (nonspecific) and adaptive immune systems, or (ii) regulation of the commensal gut microbiota and subsequent immune system molding [18]. However, there is no information available regarding the effects of dietary TC on catfish immune responses. Innate and adaptive immune parameters are important indicators of fish health status and can be used to determine the effect of feed additives on fish [19,20]. Immune responses of catfish against bacterial infection involve a series of innate and specific immune factors [21].

The objective of this study was to evaluate the immune responses of catfish fed a diet supplemented with TC. We hypothesized that TC may strengthen the immune responses of catfish such that they are better able to combat bacterial infections. This study was conducted to evaluate the expression of 13 immune-related genes in the liver, spleen, and anterior kidney (AK) in response to dietary TC supplementation in catfish. The analyzed genes included proinflammatory cytokines (interferon [IFN]-γ, interleukin [IL]-1β, and tumor necrosis factor [TNF]-α), chemokines (IL-8), and innate Toll-like receptor (TLR) genes (TLR4 and TLR5). In addition, T and B cell-related genes were analyzed, including clusters of differentiation genes (CD4-1, CD4-2, CD8-α, and CD8-β), major histocompatibility genes (MHCI and MHCII), and immunoglobulin M (IgM). Understanding the ability of TC as a feed additive to boost catfish innate and adaptive immunity will enable us to develop new tools to improve fish health and control disease outbreaks.

## 2. Material and Methods

### 2.1. Animal Ethical Oversight

Fish experiments were conducted at Mississippi State University in accordance with the Institutional Animal Care and Use Committee (IACUC # 19–388) guidelines.

### 2.2. Fish Management

A total of 100 specific pathogen-free (SPF) channel catfish fingerlings (43.5 ± 3.8 g, 15.5 ± 1.2 cm) were obtained from the College of Veterinary Medicine fish hatchery at Mississippi State University. Fish were hatched indoors and kept in facilities designed with strong biosecurity protocols to avoid infection. Fish were randomly stocked into ten 40 L tanks (10 fish/tank) supplied with flow-through dechlorinated water (flow rate: one liter per minute). Prior to the initiation of the experiment, fish were allowed one week of acclimation at 27 ± 1 °C. Water quality parameters including temperature, dissolved oxygen, and chlorine levels were monitored daily using a thermometer, meter stick, and DPD 1R tablets throughout the study.

### 2.3. Analyses of Immune-Related Gene Expression in Catfish Fed Supplemental TC

Following acclimation, fish tanks were randomized into two groups of five replicate tanks for each group. Fish in the control group were fed a commercial diet. Fish in the TC treated group were fed a commercial diet supplemented with TC (Sigma Aldrich, St. Louis, MO, USA) at a dose of 20 mg/kg feed. TC at this concentration is known to inhibit the growth of *E. ictaluri* 93–146, *F. columnare* 94–081, and *A. hydrophila* ML09–119 strains under in vitro conditions, and it also protects channel catfish from *E. ictaluri* infection [12]. The incorporation of TC into the commercial catfish diet was performed as previously described [12]. TC was extensively mixed with feeds for 10 min and a Hobart meat grinder was used to pelletize the TC feed. Before use, the feed pellets were dried in an oven for one hour. With the exception of TC, the composition of both diets was the same, as shown in Table 1. All fish were fed twice daily at the rate of 2% initial body weight for 10 days. On days 1, 7, 14, and 21 of feeding, two fish from each tank were euthanized using tricaine methane sulfonate (MS-222) overdose (300 mg/L) in accordance with the American Veterinary Medical Association (AVMA) guidelines and recommendations on euthanasia [22,23]. Spleen, liver, and anterior kidney (AK) tissues from both groups were collected aseptically and preserved in RNAlater (Thermo Fisher Scientific, Carlsbad, CA, USA) solution at 4 °C overnight. Tissues from 10 individual fish were pooled and used as one replicate.

### 2.4. Analyses of Immune-Related Gene Expression in Catfish Fed TC following E. ictaluri Challenge

*E. ictaluri* 93–146 was used for experimental infections. The strain was grown using brain heart infusion (BHI) agar and broth (Difco, Sparks, MD, USA) and incubated at 30 °C, as these are considered the optimum growth conditions for *E. ictaluri*. A total of 100 SPF channel catfish fingerlings were stocked into ten tanks (10 fish/tank). These tanks were randomized into two groups, each with 5 replicate tanks per group. The first group received a basal commercial diet (control group), and the second group received a TC-supplemented diet (treatment, TC) at 20 mg/kg diet. Fish were fed twice daily at 2% of body weight. Fish in both groups were experimentally infected with *E. ictaluri* 93–146 using bath immersion for 1 h with approximately 10^7^ CFU/mL of water as previously described [24]. Experimental TC feeding was initiated 24 h after infection and continued for 10 consecutive days after *E. ictaluri* challenge in the TC group. Ten days of TC application demonstrated its ability to enhance the survival of catfish challenged with *E. ictaluri* 93–146 [12]. Ten fish per group (two fish/tank) were euthanized at 1, 7, 14, 21, and 28 days post-infection (DPI) using MS-222. Spleen, liver, and AK tissue samples from both groups were collected aseptically and preserved in RNAlater. Tissues from 10 individual fish were pooled and used as one replicate followed by storage at −80 °C until further analysis.

### 2.5. RNA Isolation and cDNA Synthesis

Total RNA was extracted from all the tissue samples using FastRNA^TM^ SPIN kit for Microbes (MP Biomedicals, LLC, Solon, OH, USA) according to the manufacturer’s instructions. To eliminate contaminating genomic DNA, the extracted RNA was treated with RNase–free DNase I (Qiagen, Hilden, Germany). The quantity and purity of the extracted RNA were determined using a NanoDrop ND-1000 Spectrophotometer (Thermo Scientific, Waltham, MA, USA), and samples were stored at −80 °C until use. A total of 1–2 μg of RNA was used for cDNA synthesis using Maxima First Strand cDNA Synthesis Kit for RT-qPCR (Thermo Scientific; #K1641), with a final reaction volume of 20 μL. The cDNA product was diluted 50 times using DNase- and RNase-free water and used as a template for RT-qPCR.

### 2.6. RT-qPCR Analysis

The expression profiles of the immune-related TLR4, TLR5, IL-1β, IL8, TNF-α, IFN-γ, CD4-1, CD4-2, CD8α, CD8β, MHCI, MHCII, and IgM genes were determined using the FastStart Universal SYBR Green Master Mix with ROX as an internal passive reference dye as directed with 7500 Real-Time PCR System (Applied Biosystems, CA, USA) and the built-in analytical software (v 2.02). Briefly, 5 µL cDNA template was added to 0.6 µL of each primer (10 µM), 10 µL of SYBR Green Master Mix, and 3.8 µL of PCR grade water to a final volume of 20 µL. 18S rRNA was used as the reference control gene. Gene-specific primers were designed using the Primer 3 (version 4.1.0) software (Table 2). RT-PCR reactions were performed in duplicate for each sample in 96-well plates using the following thermocycling conditions: 95 °C for 10 min, followed by 40 cycles of 95 °C for 30 s, 55 °C for 30 s, and 72 °C for 1 min. DNA melt curve analyses performed at the end of each run confirmed a melting peak specific to the amplified target DNA. The relative gene expression levels were analyzed using the 2^−ΔΔCt^ method, and 18S rRNA was used as a reference to normalize the RNA input [25].

### 2.7. Statistical Analysis

Data were analyzed using paired Student’s *t*-tests in GraphPad Prism (10.1.2), with an alpha level of 0.05 having been used to determine statistical significance.

## 3. Results

### 3.1. Immune Responses of Catfish Fed a Diet Supplemented with TC

**Figure 1:**
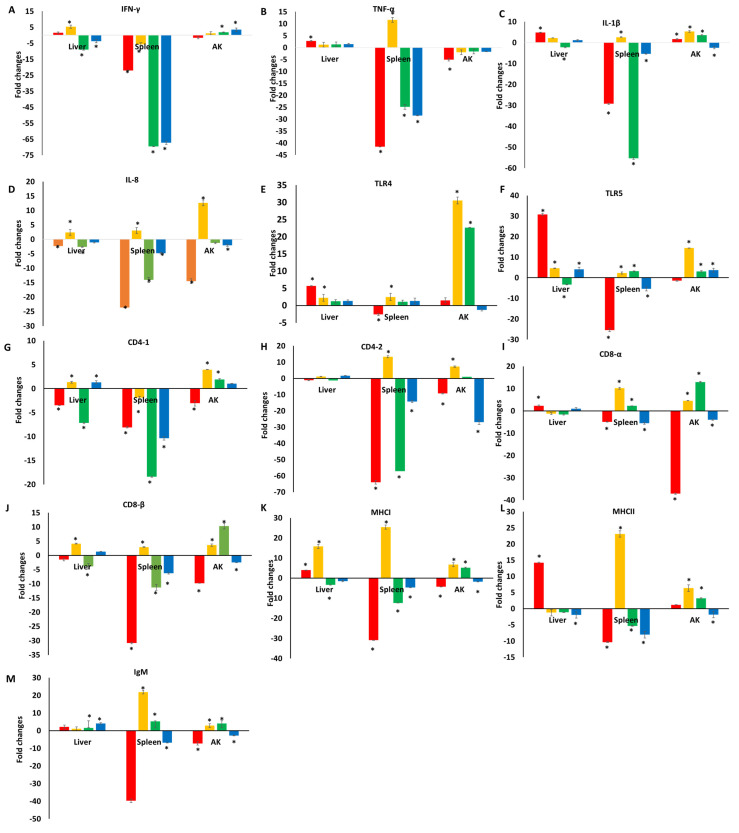
Gene expression profiles in the liver, spleen, and anterior kidney (AK) of channel catfish fingerlings that received a TC-supplemented diet as compared to those in catfish fed a normal diet on days 1 (red), 7 (orange), 14 (green), and 21 (blue). Each subfigure (**A**–**M**) represents the expression level of one gene. Data are represented as fold change in gene expression over the control group ± standard error. Four technical replicates were analyzed from a pool of ten catfish per time point. * *p* < 0.05, two-tailed Student’s *t*-test.

The expression profiles of 13 immune-related genes in the liver, spleen, and AK of channel catfish fingerlings following dietary supplementation of TC are presented in Figure 1. On the whole, significant differences in the pro-inflammatory cytokine genes expression (IFN-γ, TNF-α, and IL-1β) were observed across tissues in the TC group as compared to the control group at most time points (Figure 1A–C). Strong downregulation of all three of these genes in the spleen was evident at most analyzed time points. For example, significantly reduced IFN-γ expression was detected in the spleen of catfish fed a TC-containing diet compared to the control group on days 1, 7, 14, and 21 (21.9-, 5.6-, 69.5-, and 67.2-fold downregulation). Similarly, TNF-α was significantly downregulated on days 1, 14, and 21 (41.5-, 24-, and 28.4-fold downregulation), while IL-1β was significantly downregulated on days 1, 14, and 21 (29.2-, 55.3-, and 5.2-fold downregulation) but significantly upregulated on day 7 (2.6-fold) in the TC group. IL-8 expression was significantly decreased in the liver, spleen, and AK samples from catfish fed a TC-containing diet on days 1, 14, and 21 relatives to the control group (Figure 1D). However, a significant increase in IL-8 expression was detected on day 7 in the liver, spleen, and AK samples from catfish fed a TC-containing diet.

Although there was some variability across tissues and as a function of time, TLR4 and TLR5 tended to be upregulated in catfish in the TC treatment group as compared to controls fed a normal diet (Figure 1E,F). For instance, high levels of TLR4 upregulation were detected in AK samples from the TC group on days 7 and 14 (30.5- and 22.6-fold), while TLR5 was significantly upregulated in AK samples from these fingerlings on days 7, 14, and 21 (14.5-, 3.1-, and 3.6-fold) (Figure 1F).

Variations were similarly observed in the expression of T cell-related genes (CD4-1, CD4-2, CD8-α, and CD8-β) across time and in various tissues (Figure 1G–J). In general, these genes tended to be significantly downregulated or only modestly differentially expressed in the TC group relative to the control group, with the TC-related downregulation of these genes being most pronounced in the spleen, as all four of these genes were significantly downregulated in the spleens of TC-fed catfish on day 21 of this study.

Expression of MHC class I and class II gene expression tended to be varied over time across tissues. For instance, MHC class I was upregulated in the liver of catfish fed TC on days 1 and 7 (3.9- and 15.7-fold), while it was downregulated on days 14 and 21 (3.4- and 1.5-fold downregulation) (Figure 1K,L). However, both of these genes were substantially downregulated in the spleen days 1, 14, and 21 (MHC class I: 30.3-, 12.3-, and 4.7-fold downregulation; MHC class II: 10.3-, 5.3-, and 8.1-fold downregulation), suggesting the relatively consistent splenic suppression of the expression of these genes in response to dietary TC intake.

Patterns of IgM expression, as a measure of humoral immunity, also varied substantially across catfish tissues and time points, with significant increases in liver expression on days 1 and 21 (2.1- and 4.1-fold), whereas the opposite was observed in the spleen and AK samples at this same time point, and significant upregulation of IgM expression was observed in these tissues on days 7 (21.8- and 2.9-fold) and 14 (5.2- and 4.1-fold) (Figure 1M).

### 3.2. Immune Responses of Catfish Fed a Diet Supplemented with TC following E. ictaluri Challenge

**Figure 2:**
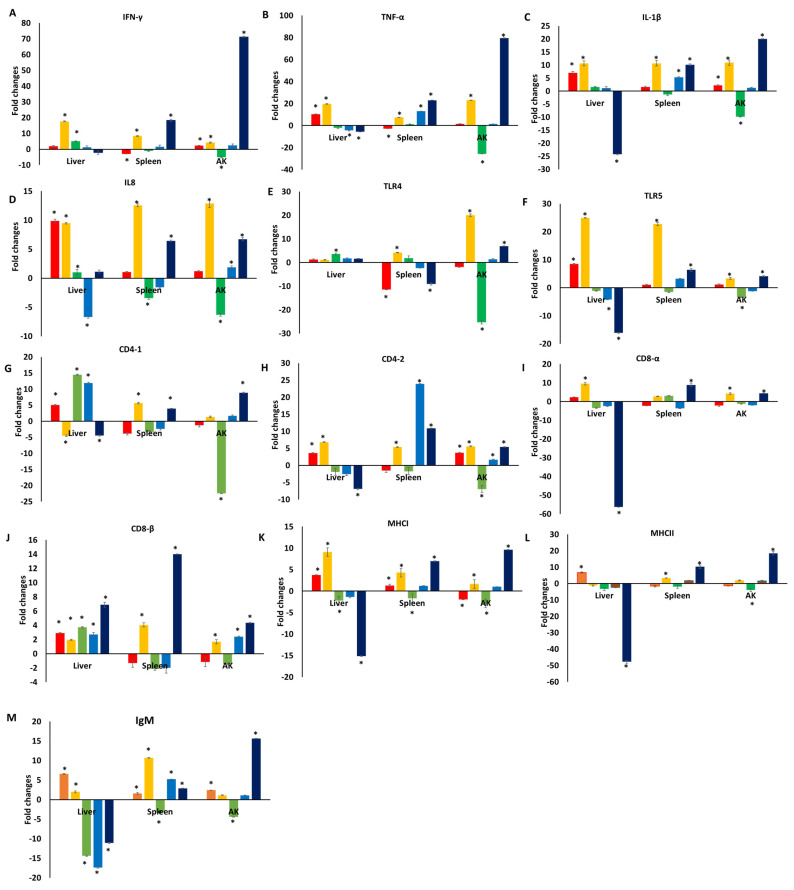
Gene expression profiles in the liver, spleen, and AK of channel catfish injected with *E. ictaluri* and fed a TC-supplemented diet as compared to catfish fed a regular diet on day 1 (red), 7 (orange), 14 (green), 21 (blue), and 28 (dark blue). Each subfigure (**A**–**M**) represents the expression level of one gene. Data are represented as fold change over the control group (received normal diet) following *E. ictaluri* challenge ± standard error. Four technical replicates were analyzed from a pool of ten tissue samples per time point. * *p* < 0.05, two-tailed Student’s *t*-test.

The levels of the proinflammatory cytokine gene (IFN-γ, TNF-α, and IL-1β) expression were generally significantly upregulated in catfish fed a TC-containing diet following *E. ictaluri* infection (Figure 2A–C). Perhaps most strikingly, all three of these genes were significantly upregulated in the spleen and AK tissue samples from catfish in the TC treatment group on day 28 as compared to the normal control group, whereas in the liver they tended to be upregulated in the TC group at early time points (days 1 and 7) but downregulated by day 28 relative to controls. In general, this same trend was also observed for IL-8 levels, although they were elevated more substantially at early time points (days 1 and 7) in the TC group across tissues, while at later time points these expression levels grew less consistent (Figure 2D).

Following *E. ictaluri* infection, enhanced TLR5 and, to a lesser extent, TLR4 expression tended to be evident in many tissues from catfish fingerlings fed a TC-containing diet at early time points (Figure 2E,F). Most notably, TLR5 was significantly upregulated in the liver of TC group fish on days 1 and 7 (8.4- and 24.9-fold), in the spleen on days 7, 21, and 28 (22.7-, 3.2-, and 6.3-fold), and in the AK on days 7 and 28 (3.3- and 4.1-fold). Changes in TLR4 expression tended to be more modest or to vary somewhat inconsistently as a function of time such that the expression patterns for this gene were less clear in response to TC intake.

While marked variability in the patterns of T cell-related gene expression (CD4-1, CD4-2, CD8-α, and CD8-β) was observed across tissues, these genes tended to be upregulated at early time points following *E. ictaluri* infection in the TC treatment group relative to normal control fish (Figure 2G–J). These effects were most pronounced for CD8-β in the liver, which was upregulated on days 1, 7, 14, and 21 (2.9-, 1.9-, 3.7-, and 2.7-fold), followed by downregulation by 6.9-fold on day 28 in the liver of catfish fed a TC-containing diet as compared to controls.

A significant increase in MHC class I and class II gene expression was observed at early time points following *E. ictaluri* infection in most analyzed tissues from catfish fed a TC-containing diet (Figure 2K,L). With time, however, a tissue-specific divergence in the expression patterns of these genes was observed such that both were strongly downregulated in the liver by day 28 post-infection despite remaining strongly upregulated in the spleen and AK samples from fish in the TC group relative to normal controls at this same time point.

A significantly enhanced IgM expression was observed in the liver of catfish fed a TC-supplemented diet on days 1 and 7 (6.5 and 1.9-fold) whereas these IL-8 levels declined significantly on days 14, 21, and 28 (Figure 2M). In contrast, IgM expression remained significantly enhanced in AK and spleen tissue samples from the TC group at all analyzed time points other than day 14, highlighting a tissue-specific difference in the expression of this humoral immunity-related gene.

## 4. Discussion

Plant-derived phytophenolic compounds, including TC, have emerged as increasingly promising alternatives to antimicrobials for both animal production and aquaculture applications [28,29]. TC is extracted from the bark of cinnamon (*Cinnamomum zeylandicum*), and as a food additive it is classified as GRAS (generally recognized as safe) by the U.S. Food and Drug Administration (approval TC-21CFR182.60) [30]. It is important to note that our prior research revealed that catfish fed a TC-containing diet at 20 mg/kg for 10 days exhibited reduced mortality relative to catfish fed a diet without TC following challenge with *E. ictaluri* (34.4% vs. 88.8%), implying that TC enhances the resistance of catfish to *E. ictaluri* infection [12]. Furthermore, mean bacterial loads in the spleen and AK were lower in fish fed TC than in fish fed a regular diet, while another study documented that a trans-cinnamic acid diet significantly improved phagocytic activity, respiratory burst activity, and the potential killing of rainbow trout phagocytic cells against *Yersinia ruckeri* [19]. No studies to date, however, have clarified the mechanisms through which TC is capable of enhancing catfish immunity. In this study, we analyzed the expression profiles of key immune-related genes in three tissues from catfish fed a diet supplemented with TC in the presence or absence of *E. ictaluri* infection, ultimately revealing that dietary TC intake may function in part by priming innate and adaptive immune responses in channel catfish such that they are better able to combat infection with this economically significant pathogen. To the best of our knowledge, this is the first report describing the effects of TC on catfish immune immunity.

To clarify the effects of TC on different facets of catfish immunity, we characterized the expression profiles of a series of proinflammatory cytokines (IFN-γ, IL-1β, and TNF-α), chemokines (IL-8), pattern-recognition receptors (PRRs; TLR4 and TLR5), MHC genes (MHCI and MHCII), and genes associated with adaptive immune responses (CD4-1, CD4-2, CD8-α, CD8-β, IgM). The initiation of immune responses in vertebrates generally stems from the initial detection of pathogen features by specific PRRs that recognize conserved pathogen-associated molecular patterns. TLR proteins are transmembrane PRRs capable of recognizing a diverse range of pathogenic moieties, initiating a robust innate immune cascade when activated [31]. Of the 17 different TLRs identified in fish [32], TLR4 and TLR5 have been suggested to serve as receptors for bacterial lipopolysaccharide (LPS) and flagellin, respectively, consistent with their reported functions in mammals [33]. However, whether TLR4 directly recognizes LPS in bony fish has been a matter of some controversy [34], with groups suggesting that TLR5, TLR25, and other receptors may also support such recognition [35]. Whatever their respective ligands, when activated, TLR4 and TLR5 engage downstream signaling pathways mediated by myeloid differentiation primary response gene 88 (MyD88), activating nuclear factor-κB (NF- κB), and thereby promoting pro-inflammatory cytokines and chemokine production [36,37]. We found that dietary TC supplementation led to significant TLR4 and TLR5 upregulation in catfish tissues. Importantly, pronounced increases in the expression of these PRRs at the mRNA level were observed during the course of *E. ictaluri* infection in the liver, spleen, and AK of catfish fed a TC-supplemented diet. This suggests that dietary TC supplementation may prime these catfish to respond to the cognate ligands more rapidly for TLR4 and TLR5, supporting the more efficient and/or more robust induction of innate immune responses to *E. ictaluri* infections. Increased TLR4 and TLR5 gene expression has previously been reported in response to the recognition of *E. ictaluri* membrane components including LPS and flagellin [21]. Similarly, the addition of the viable probiotic *Psychrobacter* sp. to the diet of *Epinephelus coioides* has been reported to promote intestinal TLR5 upregulation [38]. The observed upregulation of these two TLRs in catfish fed a TC-containing diet may help effectively engage robust innate and adaptive immune responses, particularly in the context of pathogen invasion. Other phytological extracts have been reported to exert similar benefits, as in the case of *Glycyrrhiza uralensis* extract which promoted the upregulation of TLR5 and several other TLR signaling pathway components in yellow catfish when provided as a dietary supplement, in addition to reducing cumulative mortality when these fish were challenged with *F. columnare* [39]. Interestingly, the effects of TC in this setting are in contrast to what has been reported in mammalian model systems, wherein TC has been found to suppress TLR4 expression and to blunt the engagement of inflammatory signaling downstream of TLR4 activation [40,41]. This suggests that TC may play a uniquely beneficial role in bony fish through its ability to prime innate immunity, while also emphasizing the need for further research aimed at elucidating the basis for these species-specific differences.

Pro-inflammatory cytokines (IL-1β, TNF-α, IFN-γ) induced in response to TLRs and other PRRs play a central role in the initiation of inflammatory responses, leukocyte phagocytosis, and respiratory burst activity in response to bacterial LPS or other pathogen-associated molecular patterns [42,43,44]. IL-8 is a chemokine that has been demonstrated to enhance adaptive immunity against bacterial pathogens in channel catfish when exogenously administered [45], underscoring its ability to coordinate the induction of appropriate innate and adaptive immune responses to pathogens. In this study, dietary TC application had no consistent effect on the expression of these proinflammatory cytokine genes, suggesting that TC alone does not possess any intrinsic inflammatory effects, failing to readily engage any deleterious inflammatory signaling activity. In contrast, the expression of these proinflammatory cytokines was significantly enhanced in TC-fed catfish infected with *E. ictaluri* relative to control infected catfish at different time points, suggesting that TC facilitates the more robust induction of pro-inflammatory activity on exposure to this bacterial pathogen. A prior study reported the upregulation of IL-1β, IL-8, TNF-α, and IFN-γ in the kidneys of rainbow trout fed TC at the dose of 250 mg/kg for 60 days in the absence of infection [19]. This variation between the studies is likely attributable to differences in terms of TC dose, duration of treatment, and species-specific factors. In another study, IL-1β upregulation was observed in the liver and kidney of tilapia supplemented with a mixture of organic acids [42]. In grass carp (*Ctenopharyngodon idella*), dietary cinnamaldehyde supplementation was associated with enhanced NF-κB signaling and the upregulation of certain cytokines in the intestines in fish challenged with *Aeromonas hydrophila*, although IL-1β and TNF-α were both downregulated under these conditions [46]. The effects of TC on inflammatory activity are thus likely to be highly dependent on the specific tissues, fish species, and pathogens being analyzed, emphasizing the need for more granular research focused on the underlying mechanisms.

To better probe the effects of dietary TC intake on adaptive immune response induction in channel catfish, we further analyzed the expression of MHCI and MHCII genes, as well as the CD4-1, CD4-2, CD8α, and CD8β genes. We found that all of these genes were upregulated on day 28 in both the spleen and AK of catfish fed a TC-containing diet. Mechanistically, the MHC class I and MHC class II proteins are expressed by antigen-presenting cells, respectively allowing for the presentation of intracellular and extracellular antigenic peptides to CD8^+^ and CD4^+^ T cells, thereby initiating adaptive immunity [47,48,49]. In addition to facilitating enhanced immunoglobulin production, the treatment of leukocytes from striped catfish with a range of herbal extracts has previously been reported to enhance MHC class II gene expression [50]. The observed upregulation of MHC class I and II genes in response to TC administration in the present study may help better prime the ability of antigen-presenting cells to detect bacterial incursions, while the greater expression of CD4 and CD8 in the analyzed tissue compartments suggests the expansion of these two T cell populations. However, relatively little remains known of how TC drives these increases in adaptive immune functionality, with a prior study conducted in *Cyprinus carpio* having detected no significant changes in overall leukocyte counts in response to bactericidal TC concentrations, although specific CD4^+^ and CD8^+^ T cell populations were not examined at length [51]. While there is clearly a need for further research, it is possible that by priming the induction of a stronger T cell-mediated adaptive immune response, TC may ultimately protect catfish against *E. ictaluri* infection.

Immunoglobulin M (IgM) is the primary antibody type produced in fish, and is essential for mucosal immune responses to parasites, bacteria, and viruses in channel catfish [52,53]. Here, a pronounced increase in IgM expression was detected in the spleen and AK tissues of catfish fed a diet supplemented with TC at different time points. In line with these results, a prior study in which rainbow trout feed was supplemented with trans-cinnamic acid at 500 and 750 mg/kg observed a significant increase in kidney IgM levels on day 20 of feeding [19]. In vitro, the treatment of *C. idella* kidney cells with the TC analog 3,4,5-trimethoxy cinnamic acid (10 mg/L) led to an increase in IgM expression at 48 h post-treatment [54], and increased IgM levels were observed in the gut of *C. idella* fed a range of dietary cinnamaldehyde doses and challenged with *A. hydrophilla* for 14 days [46]. In addition to TC, other feed additives including probiotic bacteria *Lactobacillus rhamnosus* in rainbow trout feed have been demonstrated to enhance renal and splenic IgM expression in bony fishes [55]. The feeding of *G. uralensis* extracts to yellow catfish also enhanced IgM expression in kidney and gill tissues, in addition to enhancing their *F. columnare* resistance as discussed above [39]. In our study, the observed TC-induced upregulation of IgM in channel catfish suggests that this dietary supplement can engage more robust cellular and humoral immune responses to *E. ictaluri*, better shielding these fish from pathogen invasion.

In summary, our results reveal that dietary TC supplementation can augment the immune status of catfish fingerlings through the basal upregulation of both PRRs necessary for the initiation of innate immunity without any corresponding inflammatory activity. In response to bacterial infection, TC intake was associated with the more robust induction of inflammatory and adaptive immunity-related gene expression, suggesting that this phytophenolic product can help protect against *E. ictaluri* infection at least in part by augmenting the ability of channel catfish to engage more rapid and robust immunological defenses upon bacterial incursion. As boosting immune status is among the most promising approaches to controlling disease and improving the overall health status of catfish, TC has great potential for use as an alternative to traditional antibiotics in aquaculture settings.

## Figures and Tables

**Table 1:** Composition of commercial catfish feed used in this study.

Diet Composition	Percent in Control Feed	Ingredients
Crude protein	32.0%	Soybean meal
Crude fat	2.5%	Rapeseed meal
Crude fiber	7.0%	Corn gluten meal
Phosphorus	0.4%	Fish meal
		Wheat shorts
		Rapeseed oil
		Mineral premix
		Sodium chloride
		Vitamin premix

**Table 2:** RT-qPCR primers for immune-related genes.

Genes	Accession NO.	Primers	References
18S ribosomal RNA	AF021880	F-GAGAAACGGCTACCACATCC R-GATACGCTCATTCCGATTACAG	[26]
CD4-1	DQ435305	F-GATGTCATCATTGTAGATCTCG R-GAGGTAGCTGGCATTTCACTCC	[27]
CD4-2	DQ435304	F-CTGTATGTTGTATCAGCCTCTG R-CAGTCACCTCCTTACTTTGGCTA	[27]
CD8-α	HQ446239	F-CTACGCGGAGAGACAGTCCCAA R-CTCACAACCCAAAAGCACATC	[27]
CD8-β	HQ446240	F-CCATCAGGCCTGGAGAAAGCA R-TCACCACCAGGAGTAGGACA	[27]
IL-1β	DQ157743	F-TGATCCTTTGGCCATGAGCGGC R-AGACATTGAAAAGCTCCTGGTC	[27]
TLR-4	x79482	F-ACCTGACTACCACACCCATA R-TCCTAGACGAGTGGAGGTTATT	This study
TLR-5	x79482	F-GGAAGCGCTACAAATCCTACT R-GTATGCCAGATCAAGTCGTATCA	This study
INFγ	NC_030434	F-TTGGGCAAAGTAGAGGACACC R-TGTTTCCACACTGCCTGTTCG	[27]
MHC class II	AF103002	F-GACACCAGGACATGGGAGGTG R-CGAGGAAGAAAGTTCCGGTAG	[27]
MHC class I I	AF103	F-GACCGAGAACTCGACTACTACA R-GAGTGCCTTTCTCCCAGTAATC	This study
TNFα	AJ417565	F-GCACAACAAACCAGACGAGA R-TCGTTGTCCTCCAGTTTCAA	[27]
IgM	x79482	F-AAGAAGCGAGTTATGCACCAG R-ATGCTTCATGTTCCACCTCAC	This study

## Data Availability

The data that support the findings of this study are available on request from corresponding author.
